# Efficacy of Serum BDNF for the Evaluation of Depressive Neurological Symptoms in Patients with Refractory Ulcerative Colitis

**DOI:** 10.3390/jcm14030874

**Published:** 2025-01-28

**Authors:** Kei Moriya, Shinsaku Nagamatsu, Yuya Nishio, Yusuke Komeda, Shoma Kikukawa, Kyohei Matsuura, Hideki Matsuo, Masakazu Uejima, Takamichi Kitagawa, Fumihiko Nakamura

**Affiliations:** 1 Department of Gastroenterology, Nara Prefecture General Medical Center, Nara 630-8581, Japan; joe.montana.no16@gmail.com (S.N.); m12082yn@jichi.ac.jp (Y.N.); yusuke9827@gmail.com (Y.K.); ks50205020@gmail.com (S.K.); graynurseshark07@kcn.jp (K.M.); h.matsuo@nara-hp.jp (H.M.); 2 Department of Endocrinology and Metabolism, Nara Prefecture General Medical Center, Nara 630-8581, Japan; m3m16d333@yahoo.co.jp; 3 Department of Laboratory Medicine, Nara Prefecture General Medical Center, Nara 630-8581, Japan; cp.ktgw@gmail.com (T.K.); fumihiconakamura@gmail.com (F.N.)

**Keywords:** brain-derived neurotrophic factor (BDNF), depression, filgotinib, Inflammatory Bowel Disease Questionnaire (IBDQ), ulcerative colitis (UC)

## Abstract

**Background/Aims:** Numerous patients with ulcerative colitis (UC) become mentally unstable after experiencing a long-standing, physically painful life, and their long-term prognosis is poorer than that of those who are mentally stable. The current study aimed to evaluate serum biomarkers for predicting mental instability, which is challenging to objectively quantify. **Methods:** In total, 29 refractory UC patients newly treated with filgotinib underwent measurements of blood parameters associated with depression and a quantitative assessment of quality of life using the Inflammatory Bowel Disease Questionnaire (IBDQ) before and after treatment initiation with a 12-week interval. The data collected were examined in relation to each other. **Results:** The induction of remission treatment with filgotinib resulted in a clinical response rate of 89.7% and a clinical remission rate of 86.2%, with all eight extraintestinal manifestations resolved. No adverse events were observed. The serum zinc, high-density lipoprotein cholesterol, mature brain-derived neurotrophic factor (BDNF) concentrations, and the IBDQ psychiatric subscores increased significantly after treatment (*p* < 0.05). Among these parameters, the mature-BDNF concentration and the IBDQ psychiatric subscore had the strongest positive correlation (R = 0.29, *p* = 0.08). Based on the logistic regression analysis, the mature-BDNF concentration (cutoff value: 20.5 ng/mL) had a sensitivity of 68.2%, specificity of 64.7%, and area under the curve of 0.67 for predicting psychiatric remission (subscore > 42.5) (*p* = 0.04). **Conclusions:** While it is not easy to objectively predict the degree of psychiatric instability in patients with refractory UC, serum mature-BDNF levels can be a useful biomarker.

## 1. Introduction

In recent years, the number of patients with ulcerative colitis (UC) has evidently increased worldwide. Moreover, the need to cooperate with each other and make concerted efforts to overcome this disease in this century is a major medical challenge [1,2]. UC is mainly characterized by inflammatory changes in the colorectal mucosa. Thus, the general consensus is that this disease is confined to the colon. However, UC is often accompanied by various extraintestinal manifestations (EIMs) such as arthritis, refractory skin diseases, and serious ophthalmologic diseases. According to recent reviews, the cumulative complication rate of all EIMs is approximately 50% [3,4,5]. In addition, in patients with EIMs, remission is more challenging to achieve due to the high disease activity of UC. In addition, these patients are at risk of relapse even after remission is achieved [3].

Based on these data, the ultimate goal of treat-to-target in UC is to improve quality of life (QoL) in addition to mucosal healing [6]. Currently, several promising compounds that can be used for the treatment of UC have been developed, and effective therapeutic agents are gradually becoming available [7]. In this context, there are numerous reports on the improvement of QoL and physical manifestations such as enteritis symptoms [8,9]. However, compared with healthy people, patients with inflammatory bowel disease (IBD), including UC, have decreased mental health even after achieving clinical remission [10]. Therefore, it is important to improve intestinal tract-related symptoms such as abdominal pain, diarrhea, and bloody stools as quickly and effectively as possible. But it is also extremely important that essential IBD treatments do not significantly affect the daily life of patients while improving various EIMs that occur in different body parts such as the joints and skin. Biologic agents that are used for the management of moderate to severe UC have various issues, such as administration time, injection site reaction, infusion reaction [11], and secondary ineffectiveness caused by the production of anti-drug antibodies [12]. In these situations, novel Janus kinase 1 selective inhibitors (JAK1-Is), which are utilized for the treatment of UC (one tablet once a day), have been available since 2022 [13,14].

Currently, filgotinib and upadacitinib are the two JAK1-I drugs available in Japan. Nevertheless, they differ not only in terms of metabolic pathways and use in combination with immunomodulators, but also in the target cytokines that produce their effects [15]. Filgotinib has a modest therapeutic effect compared with other Janus kinase inhibitors (JAK-Is) such as upadacitinib and tofacitinib, and it is also safer to use [15]. However, studies on the benefits of filgotinib in actual clinical practice are limited, and there are no reports on the development of an ideal serological index that can predict mental health recovery in patients with UC.

Therefore, this study first assessed the clinical benefit of filgotinib in patients with autologous UC and quantitatively evaluated QoL changes before and after the therapeutic intervention. To explore serological indices that can noninvasively and objectively assess and estimate the degree of psychological injury in patients with UC, we then focused on brain-derived neurotrophic factor (BDNF) [16,17,18], zinc [19], high-density lipoprotein (HDL) cholesterol [20], and hydroxylated vitamin D [21], all of which have been significantly associated with the development of psychiatric depressive symptoms in other diseases. Furthermore, the potential use of these indices as serum biomarkers was evaluated.

## 2. Methods

### 2.1. Study Population and Data Collection

Among the patients who visited our institution between May 2022 and December 2023 and who were diagnosed with UC clinically and endoscopically using the diagnostic criteria outlined by the Ministry of Health, Labor and Welfare in Japan (http://www.ibdjapan.org/pdf/doc15.pdf [Only Japanese text available]. Accessed on 23 November 2024), 30 voluntarily enrolled in the current study. One patient who had a slowly progressive renal dysfunction and who was not eligible for treatment with filgotinib, which is mainly metabolized by the kidney, based on the physician’s discretion was excluded from the analysis. Subsequently, 29 patients with UC who did not present with hepatitis B, C virus, human immunodeficiency virus, and tuberculosis infections were included in this analysis. All participants were treated additionally with filgotinib (200 mg per day) during the observation period (12 weeks).

### 2.2. Laboratory Assessments

Blood tests were performed at induction baseline (week 0) and maintenance baseline (week 12). The serum zinc, copper, 25-hydroxy vitamin D, and leucine-rich alpha 2-glycoprotein concentrations were measured by BML Inc. (Tokyo, Japan) using atomic absorption spectrophotometry, colorimetry, electrochemiluminescence immunoassay, and latex agglutination immunoassay, respectively. The serum BDNF levels were measured in duplicate using the following commercial enzyme-linked immunosorbent assay (ELISA) kits according to the manufacturer’s instructions: human BDNF ELISA kit #BEK-2211 mature-BDNF and #BEK-2237 pro-BDNF (Biosensis^®^, Thebarton, SA, Australia). During the pro-BDNF measurement, all samples were diluted with a heterophilic antibody-blocker (BL-004-500) to reduce the effect of the heterophilic antibody, which then decreases the false-positive optical density values. The patients underwent routine laboratory examination, which included complete blood count and the general biochemistry test.

### 2.3. Clinical Efficacy Assessments

A clinically effective case was defined as a case in which the partial Mayo score (pMS) reduced by at least 2 and by at least 30% compared with the pretreatment level. Clinical remission was defined as a pMS ≤ 2 plus individual subscores ≤ 1. Clinical relapse was defined as the need for treatment modification or intensification due to worsening of the clinical symptoms of UC.

### 2.4. Quality of Life Assessments

The QoL of patients with UC was quantically and sequentially evaluated using the Inflammatory Bowel Disease Questionnaire (IBDQ), which had been already validated [22]. The total IBDQ score was used to assess disease-specific health-related QoL at the induction baseline and maintenance baseline (12 weeks). The IBDQ comprises 32 items in 4 domains (bowel conditions, systemic conditions, psychiatric conditions, and social aspects) [22]. The total score for the 32 items ranges from 0 to 224, with higher scores indicating a better health-related QoL [22]. The minimal clinically important difference was defined as a 16-point increase in the total IBDQ score from the induction baseline [23]. Remission was defined as a total IBDQ score ≥ 170 [24]. The quantitative QoL assessment using the IBDQ was conducted after obtaining permission from the Industry Liaison Office at McMaster University.

### 2.5. Statistical Analyses

Numerical variables were presented as the median with interquartile ranges. Unrelated categorical variables were assessed using Pearson’s chi-square test. The Mann–Whitney U test was adopted to examine significant differences between two groups. Correlation was evaluated using the Spearman’s rank correlation coefficients. *p* values of <0.05 indicated statistically significant differences. The JMP version 14.3.0 (SAS Institute Inc., Cary, NC, USA) software was used for statistical analyses.

### 2.6. Ethical Issues

The current study was conducted according to the principles of the Japanese ethics guideline for life science and medical research involving human subjects (https://www.mext.go.jp/lifescience/bioethics/files/pdf/n2373_01.pdf [Only Japanese text available]. Accessed on 23 November 2024) and the Declaration of Helsinki. Further, it was approved by the Ethical Committee of Nara Prefecture General Medical Center (approval number: #887). Informed consent of this study was obtained by an opt-out method.

## 3. Results

### 3.1. Clinical Characteristics of the Study Population

Table 1 shows the clinical profiles of 29 patients with UC at the induction baseline. The median age of the patients was 41.0 years, and the male-to-female ratio was 17:12. Approximately 58.6% (n = 17) of the patients presented with the pancolitis type and 41.4% (n = 12) with the left-sided colitis type. None of the patients had the proctitis type. Further, from the viewpoint of disease severity classification, 10.3% (3/29), 86.2% (25/29), and 3.4% (1/29) of the patients exhibited mild, moderate, and severe disease, respectively. Eight (27.6%) patients exhibited EIMs. Among them, seven had arthritis and one had pyoderma gangrenosum. Corticosteroids and thiopurine were used as a concomitant drug in 31.0% (n = 9) and 34.5% (n = 10) of the patients, respectively. According to the number of advanced therapies including biologics, JAK-I, and calcineurin inhibitor, 24.1% (n = 7) of the patients had been treated with one agent and 20.7% (n = 6) with two. All of them received continuous treatment with the addition of filgotinib until week 12.

### 3.2. Objective Efficacy of Filgotinib in the Study Population

**Figure 1:**
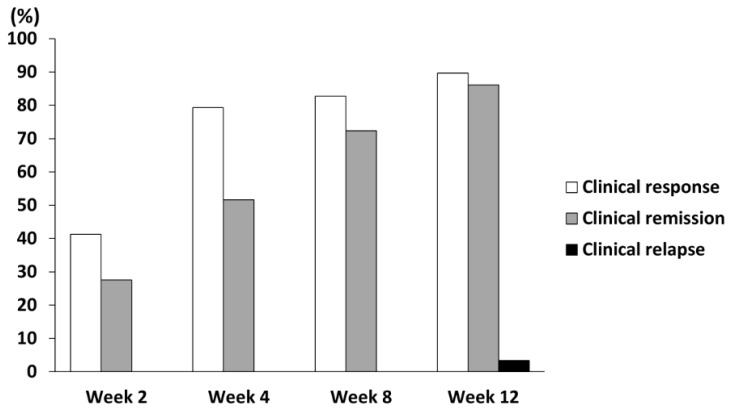
Proportions of patients treated with filgotinib (200 mg per day) who achieved clinical response, clinical remission, and clinical relapse.

As shown in Table 2, the disease severity significantly improved after treatment with filgotinib, and all EIMs disappeared. The concomitant use of corticosteroids became unnecessary in all cases, and the median pMS decreased from 5.0 to 1.0 after filgotinib intervention for 12 weeks. The blood test indices including white blood cell counts, neurocyte counts, neutrophil-to-lymphocyte ratio, platelet count, C-reactive protein, and leucine-rich alpha 2-glycoprotein, which are associated with intestinal inflammation, significantly decreased. The 12-week clinical response rate was 89.7% (n = 26), and the clinical remission rate was 86.2% (n = 25) (Figure 1). Only one male patient presented with clinical relapse at week 12 (3.4%). Among the items used as potential novel biomarkers associated with changes in disease status, the serum HDL cholesterol and zinc concentrations significantly increased (*p* < 0.01). However, the 25 hydroxy vitamin D levels did not significantly change. The mature-BDNF concentration exhibited an evidently increasing trend (*p* = 0.053). Meanwhile, the pro-BDNF levels were extremely low compared with the mature-BDNF levels and did not increase significantly before and after treatment (*p* = 0.158).

### 3.3. Subjective Efficacy of Filgotinib in the Study Population

**Figure 2:**
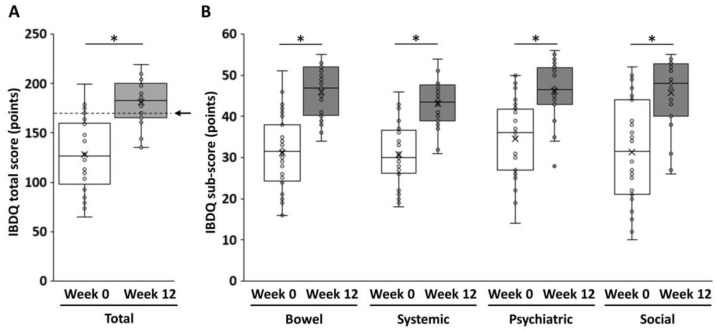
Changes in the IBDQ score before and after filgotinib induction. (**A**) The total IBDQ score and (**B**) the IBDQ subscores. The arrow indicates the minimum level of IBDQ remission (170 points). The Mann–Whitney U test was adopted to examine significant differences between two groups. *p* values of <0.05 indicated statistically significant differences. *, *p* < 0.05. IBDQ, Inflammatory Bowel Disease Questionnaire.

The subjective efficacy of filgotinib was evaluated using IBDQ at the induction baseline and maintenance baseline with a 12-week interval. The median total IBDQ score at the induction baseline was 127.0 (interquartile range: 102.0–159.3), and it increased to >50 at week 12 (median: 183, interquartile range: 167.3–198.8) (*p* < 0.05) (Figure 2A). The subscores for bowel conditions, systemic conditions, psychiatric condition, and social aspects increased significantly after the treatment. The subscores for bowel conditions had the highest average increase (Δ14.9 points). Meanwhile, the subscores for psychiatric conditions had the lowest average increase (Δ11.5 points) (Figure 2B).

### 3.4. Association Between the Psychiatric IBDQ Subscore and Serum Biochemical Indices

**Figure 3:**
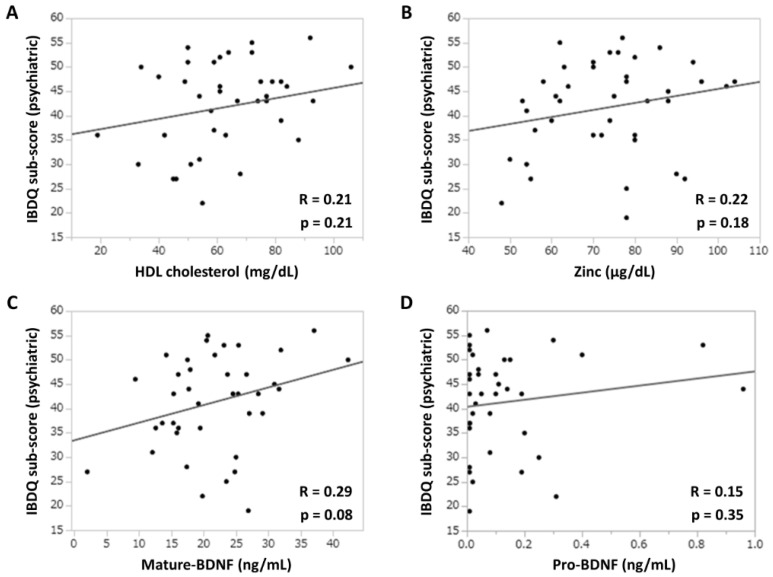
Association between the IBDQ psychiatric subscores and depression-related clinical parameters. (**A**) IBDQ subscore and serum HDL (mg/dL) concentration. (**B**) IBDQ subscore and serum zinc (μg/dL) concentration. (**C**) IBDQ subscore and serum mature-BDNF (ng/mL) concentration. (**D**) IBDQ subscore and serum pro-BDNF (ng/mL) concentration. Correlation was evaluated using Spearman’s rank correlation coefficients. *p* values of <0.05 indicated statistically significant differences. BDNF, brain-derived neurotrophic factor; HDL, high-density lipoprotein; IBDQ, Inflammatory Bowel Disease Questionnaire.

**Figure 4:**
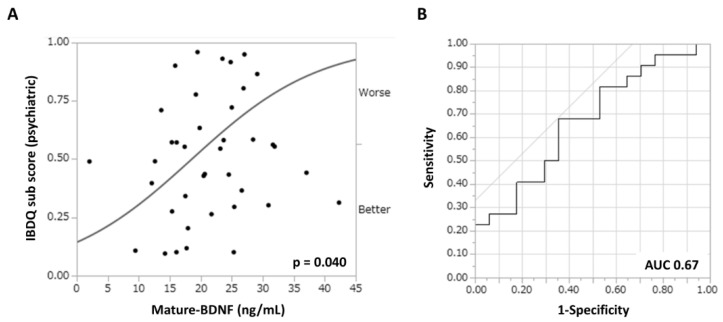
Efficacy of the mature-BDNF concentration in predicting depressive neurological symptoms (IBDQ psychiatric subscores < 42.5) in patients with refractory UC. ROC curve analysis. (**A**) Bivariate analysis between the serum mature-BDNF (ng/mL) concentration and the IBDQ subscore (points). (**B**) ROC curves for depressive neurological symptoms (AUC = 0.67, sensitivity = 68.2%, and specificity = 64.7%). *p* values of <0.05 indicated statistically significant differences. AUC, area under the curve; BDNF, brain-derived neurotrophic factor; ROC, receiver operating characteristic.

Figure 3 shows the association between depressive neurological symptoms and serum biomarker levels in patients with UC treated with filgotinib. The correlation coefficients (r) between the IBDQ psychiatric subscore and serum HDL cholesterol or zinc concentration were 0.21 and 0.22, respectively (Figure 3A,B). By contrast, the correlation coefficient (r) between the mature-BDNF concentration and the IBDQ psychiatric subscore was 0.29 (*p* = 0.08), which was higher than that of the previous two items (Figure 3C). However, there was no significant association found between pro-BDNF concentrations and the IBDQ psychiatric subscore, with a correlation coefficient (r) of 0.15 (Figure 3D). To examine the efficacy of mature-BDNF concentrations for the evaluation of depressive neurological symptoms, a logistic regression analysis was performed. Results showed that the mature-BDNF levels (cutoff value: 20.5 ng/mL) had a sensitivity of 68.2%, specificity of 64.7%, and area under the receiver operating characteristic of 0.67 for predicting psychological remission (IBDQ psychiatric subscore > 42.5) (*p* = 0.040). The cutoff value of 20.5 ng/mL for mature-BDNF was calculated along with the sensitivity analysis (Figure 4A,B).

## 4. Discussion

In the current study, the serum BDNF concentration [16,17,18] was found to be most strongly correlated with mental instability in patients with refractory UC, even when compared with the serum zinc [19], HDL cholesterol [20], and hydroxy vitamin D levels [21], which have been previously reported to be associated with mental depression. BDNF, the most major neurotrophic factor in the brain and first purified from the porcine brain in 1982, is strongly expressed in excitatory neurons mainly in the hippocampus and cerebral cortex. Moreover, it has effects such as maintaining neuronal survival and inducing differentiation [25,26,27,28,29]. Furthermore, BDNF is involved in neural plasticity based on neural activity [30]. BDNF is synthesized as a precursor, pro-BDNF, and then processed to become mature-BDNFs with a molecular weight of 13.5 kDa [31], which are then released from the presynaptic vesicles in response to stimuli and recognized by postsynaptic receptors for signaling [32,33]. Furthermore, BDNF has central appetite suppression effects [34] and is associated with diabetes mellitus and associated psychiatric and neurological symptoms [35,36]. Several studies have investigated the association between human serum BDNF levels and psychiatric disorders [16,17,18]. Although serum BDNF is believed to be platelet-derived, its own significance is unresolved. However, since the methylation status of BDNF in the brain and blood is correlated based on animal studies [37], it is assumed that the serum BDNF levels reflect the brain BDNF levels in humans. Some studies have shown that the BDNF protein expression is decreased in the postmortem brains of patients with depression and those who committed suicide, and reports showing increased DNA (deoxyribonucleic acid) methylation of the BDNF gene promoter in their peripheral blood mononuclear cells also support the aforementioned notions [38,39]. In addition, an extremely interesting animal study reported that a serotonin reuptake inhibitor, which is used for depression, increased the BDNF expression in the mouse brain [40]. Based on the abovementioned information, the serum BDNF concentration is positively associated with the BDNF expression in the brain and may be a good biomarker that can reflect the ups and downs of a patient’s psychiatric symptoms. Patients with UC have a high rate of diverse and major disease-related events during the long-term clinical course after the diagnosis is confirmed [41]. It is easy to conclude that their mental instability is more intense than that of healthy individuals. In addition, patients with UC who are in a psychologically vulnerable situation have significantly higher rates of apparent disease flare-ups, hospitalization, and disease-related mortality, compared with patients who are mentally stable [42]. The difference in the incidence of these adverse events is even higher when remission is not achieved, due to a lack of treatment response, resulting in prolonged clinical disease activity [42]. Based on these facts, there is no doubt that the ability of gastroenterologists, who generally do not specialize in the treatment of psychiatric disorders, to objectively evaluate depression in patients with UC using quantitative indices can be significantly beneficial in terms of significantly improving the overall QoL of patients with UC.

While a large number of cytokines, such as interferon-gamma, tumor necrosis factor (TNF)-alpha, interleukin (IL)-1beta, IL-5, IL-6, IL-13, IL-17A, and IL-23 are involved in the pathogenesis of UC [43], the expression levels of TNF-alpha, IL-5, IL-6, IL-17A, and IL-23 among these cytokines have been reported to be significantly increased in depressed patients compared to healthy controls [44,45]. In addition, the presence of prolonged stress is thought to contribute to the development of depression by increasing the permeability of the blood–brain barrier, thereby allowing the entry of inflammatory cytokines into the brain [46,47]. These inflammatory cytokines cause microglial hyperactivation, neuroinflammation, and impairment of the BDNF pathway [48]. In the current study, JAK-I administered to the UC patients may have calmed intestinal inflammation by blocking the aforementioned cytokine signaling, resulting in a decrease in blood endotoxin levels by correcting the abnormal increase in intestinal permeability and restoring dysbiosis. In addition, given the fact that humans treated with pro-inflammatory substance lipopolysaccharide increased levels of pro-inflammatory cytokines in their blood, resulting in sick and depressive-like behavior [49,50], it is easy to see why JAK-I treatment can improve the depressive state of UC patients. It is also possible that the suppression of IL-6, a pro-inflammatory cytokine, by filgotinib may have contributed to the improvement of depression, but this point awaits clarification in future studies.

The current study showed that filgotinib, a JAK1-I, rapidly and reliably suppressed the disease activity in UC and was extremely effective in controlling the pathogenesis of EIM (Table 2). Although a sequential evaluation of filgotinib (pre- and post-treatment at 3-month intervals) revealed a significant improvement in the QoL of patients (Figure 2A), the quantitative evaluation using the IBDQ showed that the improvement in the psychiatric condition was minimal compared with the other three domains (bowel conditions, systemic conditions, and social aspects) (Figure 2B). This finding, in turn, indicated that UC is a condition that not only damages the patient’s intestinal mucosa, but also reflects major psychological damage, including a sense of challenge and self-esteem, which are essential to the patient’s future. It is easy to imagine that even if a patient exhibited remission in the clinical course of this disease, which is challenging to completely cure with modern medicine, it is extremely difficult to disregard the fear that the disease can flare up and can threat one’s daily life or even survival. Filgotinib is a type of small-molecular-weight compound. It is similar to tacrolimus (calcineurin inhibitor) and carotegrast methyl (alpha 4 integrin inhibitor), which are currently available in Japan for the treatment of UC. Moreover, it is classified as JAK1-I, which has a higher safety profile [15]. This drug is advantageous as it can be used without concerns about antibody production and in combination with thiopurine [15]. However, filgotinib also has some disadvantages. For example, it cannot be used in patients with renal impairment. This is because it is a small-molecular-weight compound that is excreted by the kidneys. Moreover, it is associated with an increased risk of abnormal blood test results and the development of shingles after medication initiation. There are also safety concerns with its long-term continuous use. However, the frequency of occurrence may vary as with other JAK-Is [15]. In addition, the effects of amplification or weakening of efficacy caused by the concomitant use of drugs in patients with UC (particularly the elderly) who have current polypharmacy should be considered [51]. However, filgotinib is associated with a relatively low risk of herpes zoster infection compared with other JAK-Is [15], and it had a favorable effect on EIMs such as arthritis and pyoderma gangrenosum in patients with UC, with all EIMs resolving during the observation period. Based on these facts, filgotinib can be an effective option, in addition to other biologics, as a treatment for improving the condition of patients with refractory UC.

The current study had several limitations that should be considered when interpreting the results. First, the sample size in this study was limited because it was challenging to recruit a large number of patients with refractory UC who were treated with the same therapy within a limited period. In order to further increase the reliability of the results of this study, it is desirable to launch a multicenter study in which a larger number of patients are expected to be enrolled and to conduct the analysis in the future. Second, the results of this study might be biased by the effect of circadian rhythm in the measurement of serum BDNF, zinc, and 25-hydroxy vitamin D levels [52,53,54]. Although intra- and interindividual differences could be fairly limited because the patients in this study presented at approximately the same time each visit during limited daytime office hours, efforts to align the time of blood collection as much as possible will be eagerly awaited in order to minimize differences in measurements due to circadian variations among patients. Third, because this study was limited to patients with refractory UC treated with filgotinib, it cannot be assured that similar results will be obtained in patients treated with other biologics or agents. Fourth, the wide age distribution of the patients and the disproportionate male/female ratio are considered to be additional limitations of this study. Since it is widely known that the incidence of depression is higher in middle-aged and older persons than in younger persons and in women than in men, future studies will be required to collect the number of subjects that can be analyzed by group from these perspectives.

However, despite these limitations, this study showed the usefulness of serum BDNF level as a simple and objective biomarker of psychological recovery, which had minimal improvement compared with physical symptoms in the early stage after medical treatment intervention, in patients with UC. The quantitative assessment of mental stability is still not as easy as the evaluation of physical and organ symptoms. Nevertheless, with consideration of its usefulness, further studies with a significantly larger number of patients should be conducted in the near future to increase the reliability of the results of the current study.

## Figures and Tables

**Table 1:** Baseline demographics and disease characteristics.

Age (years), median [IQR]	41.0 [28.0–62.0]
Sex (male/female), n (%)	17/12 (58.6%/41.4%)
BMI (kg/m^2^), median [IQR]	21.0 [19.5–23.0]
Smoking history, n (%)	4 (13.8%)
Drinking history, n (%)	3 (10.3%)
Past history of anti-tubercular treatment, n (%)	0 (0.0%)
Diabetes Mellitus, n (%)	1 (3.4%)
Malignant neoplasm, n (%)	0 (0.0%)
Disease duration (years), median [IQR]	2.0 [1.0–7.0]
Disease extent	
Pancolitis, n (%)	17 (58.6%)
Left-sided colitis, n (%)	12 (41.4%)
Proctitis, n (%)	0 (0.0%)
Disease type	
Relapsed, n (%)	23 (79.3%)
Persisted, n (%)	4 (13.8%)
First attack, n (%)	2 (6.9%)
Severity	
Mild, n (%)	3 (10.3%)
Moderate, n (%)	25 (86.2%)
Severe, n (%)	1 (3.4%)
EIM, n (%)	8 (27.6%)
History of treatment at baseline	
Previous corticosteroid use, n (%)	28 (96.6%)
Previous thiopurine use, n (%)	11 (37.9%)
Previous advanced therapy * use, n (%)	13 (44.8%)
Number of previous advanced therapy *	
1 agent, n (%)	7 (24.1%)
2 agents, n (%)	6 (20.7%)
3 agents, n (%)	0 (0.0%)
Concomitant use of 5-ASA, n (%)	24 (82.8%)
Concomitant use of corticosteroid, n (%)	9 (31.0%)
Concomitant use of Thiopurine, n (%)	10 (34.5%)
Partial Mayo score, median [IQR]	5.0 [5.0–6.0]

5-ASA, 5-aminosalicylates; BMI, body mass index; EIM, extra-intestinal manifestations; IQR, interquartile range. * Advanced therapy includes anti-TNF (tumor necrosis factor) antibody, anti-IL (interleukin) 12/23 antibody, anti-IL23 antibody, anti-α4β7 integrin antibody, calcineurin inhibitor, and janus kinase inhibitor (except filgotinib).

**Table 2:** Clinical parameters at baseline and 12 weeks after the start of filgotinib.

	Week 0 (n = 29)	Week 12 (n = 29)	*p*
Severity			<0.01
Mild, n (%)	3 (10.3%)	28 (96.6%)	
Moderate, n (%)	25 (86.2%)	1 (3.4%)	
Severe, n (%)	1 (3.4%)	0 (0.0%)	
EIM, n (%)	8 (27.6%)	0 (0.0%)	<0.01
Concomitant use of 5-ASA, n (%)	24 (82.8%)	24 (82.8%)	1.000
Concomitant use of corticosteroid, n (%)	9 (31.0%)	0 (0.0%)	<0.01
Concomitant use of Thiopurine, n (%)	10 (34.5%)	1 (3.5%)	<0.01
Partial Mayo score, median [IQR]	5.0 [5.0–6.0]	1.0 [0.0–2.0]	<0.01
White blood cells (/µL), median [IQR]	6800 [5350–9075]	5200 [4525–6175]	<0.01
Neutrocytes (/µL), median [IQR]	4624 [3050–7260]	2808 [2095–3631]	<0.01
Lymphocytes (/µL), median [IQR]	1564 [792–2768]	1872 [1303–2581]	<0.01
N/L ratio, median [IQR]	2.9 [1.9–5.3]	1.5 [1.1–2.0]	<0.01
Hemoglobin (g/dL), median [IQR]	12.8 [11.5–13.8]	13.4 [12.5–14.5]	0.156
Platelets (×10^4^/µL), median [IQR]	35.7 [27.8–45.3]	26.7 [24.0–32.3]	<0.01
C-related protein (mg/dL), median [IQR]	0.3 [0.1–1.2]	0.0 [0.0–0.1]	0.027
LRG (µg/mL), median [IQR]	25.7 [22.2–31.7]	9.3 [7.9–11.0]	<0.01
HDL cholesterol (mg/dL), median [IQR]	52.5 [41.5–58.3]	73.0 [61.0–81.3]	<0.01
Zinc (µg/dL), median [IQR]	70.0 [54.0–78.0]	77.5 [67.8–88.0]	<0.01
Copper (µg/dL), median [IQR]	107.0 [97.0–125.5]	89.0 [72.5–100.8]	0.068
25-hydroxy Vit.D (ng/mL), median [IQR]	16.8 [10.9–20.3]	17.0 [15.0–22.4]	0.177
Mature-BDNF (ng/mL), median [IQR]	18.6 [15.3–24.5]	23.7 [17.7–27.0]	0.053
Pro-BDNF (ng/mL), median [IQR]	0.03 [0.01–0.13]	0.07 [0.01–0.19]	0.158

5-ASA, 5-aminosalicylates; BDNF, brain-derived neurotrophic factor; EIM, extra-intestinal manifestations; HDL, high density lipoprotein; IQR, interquartile range; LRG, leucine rich alpha-2-glycoprotein.

## Data Availability

All the data used to support the findings of this study are included in the article.

## Abbreviations for the Figures

AUC	Area under the curve
BDNF	Brain-derived neurotrophic factor
HDL	High-density lipoprotein
IBDQ	Inflammatory Bowel Disease Questionnaire

## References

[1] Mak J.W.Y., Sun Y., Limsrivilai J., Abdullah M., Kaibullayeva J., Balderramo D., Vergara. B.I., Paudel M.S., Banerjee R., Hilmi I. (2023). Development of the global inflammatory bowel disease visualization of epidemiology studies in the 21st century (GIVES-21). BMC Med. Res. Methodol..

[2] Kaplan G.G., Ng S.C. (2017). Understanding and preventing the global increase of inflammatory bowel disease. Gastroenterology.

[3] Greuter T., Rieder F., Kucharzik T., Peyrin-Biroulet L., Schoepfer A.M., Rubin D.T., Vavricka S.R. (2021). Emerging treatment options for extraintestinal manifestations in IBD. Gut.

[4] Juillerat P., Manz M., Sauter B., Peyrin-Biroulet L., Schoepfer A.M., Rubin D.T., Vavricka S.R. (2020). Therapies in Inflammatory Bowel Disease Patients with Extraintestinal Manifestations. Digestion.

[5] Guillo L., D’Amico F., Danese S., Peyrin-Biroulet L. (2021). Ustekinumab for extra-intestinal manifestations of inflammatory bowel disease: A systematic literature review. J. Crohn’s Colitis.

[6] Turner D., Ricciuto A., Lewis A., D’Amico F., Dhaliwal J., Griffiths A.M., Bettenworth D., Sandborn W.J., Sands B.E., Reinisch W. (2021). STRIDE-II: An update on the selecting therapeutic targets in inflammatory bowel disease (STRIDE) initiative of the international organization for the study of IBD (IOIBD): Determining therapeutic goals for treat-to-target strategies in, I.B.D. Gastroenterology.

[7] Le Berre C., Honap S., Peyrin-Biroulet L. (2023). Ulcerative colitis. Lancet.

[8] Chavarría C., Casanova M.J., Chaparro M., Barreiro-de Acosta M., Ezquiaga E., Bujanda L., Rivero M., Argüelles-Arias F., Martín-Arranz M.D., Martínez-Montiel M.P. (2019). Prevalence and factors associated with fatigue in patients with inflammatory bowel disease: A multicentre study. J. Crohn’s Colitis.

[9] Romberg-Camps M.J., Bol Y., Dagnelie P.C., Hesselink-van de Kruijs M.A.M., Kester A.D.M., Engels L.G., van Deursen C., Hameeteman W.H., Pierik M., Wolters F. (2010). Fatigue and health-related quality of life in inflammatory bowel disease: Results from a population-based study in the Netherlands: The IBD-South Limburg cohort. Inflamm. Bowel Dis..

[10] Römkens T.E., van Vugt-van Pinxteren M.W., Nagengast F.M., van Oijen M.G., de Jong D.J. (2011). High prevalence of fatigue in inflammatory bowel disease: A case control study. J. Crohn’s Colitis.

[11] Shivaji U.N., Sharratt C.L., Thomas T., Smith S.C.L., Iacucci M., Moran G.W., Ghosh S., Bhala N. (2019). Review article: Managing the adverse events caused by anti-TNF therapy in inflammatory bowel disease. Aliment. Pharmacol. Ther..

[12] Velikova T., Sekulovski M., Peshevska-Sekulovska M. (2024). Immunogenicity and loss of effectiveness of biologic therapy for inflammatory bowel disease patients due to anti-drug antibody development. Antibodies.

[13] Feagan B.G., Danese S., Loftus E.V., Vermeire S., Schreiber S., Ritter T., Fogel R., Mehta R., Nijhawan S., Kempiński R. (2021). Filgotinib as induction and maintenance therapy for ulcerative colitis (SELECTION): A phase 2b/3 double-blind, randomised, placebo-controlled trial. Lancet.

[14] Danese S., Vermeire S., Zhou W., Pangan A.L., Siffledeen J., Greenbloom S., Hébuterne X., D’Haens G., Nakase H., Panés J. (2022). Upadacitinib as induction and maintenance therapy for moderately to severely active ulcerative colitis: Results from three phase 3, multicentre, double-blind, randomised trials. Lancet.

[15] Nakase H. (2023). Understanding the efficacy of individual janus kinase inhibitors in the treatment of ulcerative colitis for future positioning in inflammatory bowel disease treatment. Immunol. Med..

[16] Yoshida T., Ishikawa M., Niitsu T., Nakazato M., Watanabe H., Shiraishi T., Shiina A., Hashimoto T., Kanahara N., Hasegawa T. (2012). Decreased serum levels of mature brain-derived neurotrophic factor (BDNF), but not its precursor pro-BDNF, in patients with major depressive disorder. PLoS ONE.

[17] Martinotti G., Pettorruso M., Berardis D., Varasano P.A., Lucidi Pressanti G., De Remigis V., Valchera A., Ricci V., Di Nicola M., Janiri L. (2016). Agomelatine increases BDNF serum levels in depressed patients in correlation with the improvement of depressive symptoms. Int. J. Neuropsychopharmacol..

[18] Merabtine T., Tarhini Z., Preux P.M., Christou N., Jost J. (2024). Effects of antidepressant and antipsychotic medication on peripheral brain-derived neurotrophic factor concentration: Systematic review and meta-analysis. Psychiatry Res..

[19] Nishikawa H., Enomoto H., Yoh K., Iwata Y., Sakai Y., Kishino K., Ikeda N., Takashima T., Aizawa N., Takata R. (2020). Serum zinc concentration and quality of life in chronic liver diseases. Medicine.

[20] Melin E.O., Thulesius H.O., Hillman M., Svensson R., Landin-Olsson M., Thunander M. (2019). Lower HDL-cholesterol, a known marker of cardiovascular risk, was associated with depression in type 1 diabetes: A cross sectional study. Lipids Health Dis..

[21] Kaviani M., Nikooyeh B., Etesam F., Behnagh S.J., Kangarani H.M., Arefi M., Yaghmaei P., Neyestani T.R. (2022). Effects of vitamin D supplementation on depression and some selected pro-inflammatory biomarkers: A double-blind randomized clinical trial. BMC Psychiatry.

[22] Yarlas A., Maher S., Bayliss M., Lovley A., Cappelleri J.C., Bushmakin A.G., DiBonaventura M.D. (2020). The inflammatory bowel disease questionnaire in randomized controlled trials of treatment for ulcerative colitis: Systematic review and meta-analysis. J. Patient Cent. Res. Rev..

[23] Irvine E.J. (1999). Development and subsequent refinement of the inflammatory bowel disease questionnaire: A quality-of-life instrument for adult patients with inflammatory bowel disease. J. Pediatr. Gastroenterol. Nutr..

[24] Marinelli C., Savarino E., Inferrera M., Lorenzon G., Rigo A., Ghisa M., Facchin S., D’Incà R., Zingone F. (2019). Factors influencing disability and quality of life during treatment: A cross-sectional study on IBD patients. Gastroenterol. Res. Pract..

[25] Barde Y.A., Edgar D., Thoenen H. (1982). Purification of a new neurotrophic factor from mammalian brain. EMBO J..

[26] Zagrebelsky M., Korte M. (2014). Form follows function: BDNF and its involvement in sculpting the function and structure of synapses. Neuropharmacology.

[27] Numakawa T., Suzuki S., Kumamaru E., Adachi N., Richards M., Kunugi H. (2010). BDNF function and intracellular signaling in neurons. Histol. Histopathol..

[28] Lu Y., Christian K., Lu B. (2008). BDNF: A key regulator for protein synthesis-dependent LTP and long-term memory?. Neurobiol. Learn. Mem..

[29] Alster P., Madetko-Alster N., Otto-Ślusarczyk D., Migda A., Migda B., Struga M., Friedman A. (2023). Role of orexin in pathogenesis of neurodegenerative parkinsonisms. Neurol. Neurochir. Pol..

[30] Thoenen H. (2000). Neurotrophins and activity-dependent plasticity. Prog. Brain Res..

[31] Deinhardt K., Chao M.V. (2014). Shaping neurons: Long and short range effects of mature and pro-BDNF signaling upon neuronal structure. Neuropharmacology.

[32] Horch H.W., Krüttgen A., Portbury S.D., Katz L.C. (1999). Destabilization of cortical dendrites spines by, B.D.N.F. Neuron.

[33] Horch H.W., Katz L.C. (2002). BDNF release from single cells elicits local dendritic growth in nearby neurons. Nat. Neurosci..

[34] Takei N., Furukawa K., Hanyu O., Sone H., Nawa H. (2014). A possible link between BDNF and mTOR in control of food intake. Front. Psychol..

[35] Gaur A., Varatharajan S., Balan Y., Taranikanti M., John N.A., Umesh M., Ganji V., Medala K. (2024). Brain-derived neurotrophic factor (BDNF) and other neurotrophic factors in type 2 diabetes mellitus and their association with neuropathy. Ir. J. Med. Sci..

[36] He W.L., Chang F.X., Wang T., Sun B.X., Chen R.R., Zhao L.P. (2024). Serum brain-derived neurotrophic factor levels in type 2 diabetes mellitus patients and its association with cognitive impairment: A meta-analysis. PLoS ONE.

[37] Duffy H.B.D., Roth T.L. (2020). Increases in bdnf DNA methylation in the prefrontal cortex following aversive caregiving are reflected in blood tissue. Front. Hum. Neurosci..

[38] Castren E., Monteggia L.M. (2021). Brain-derived neurotrophic factor signaling in depression and antidepressant action. Biol. Psychiatry.

[39] Hing B., Sathyaputri L., Potash J.B. (2018). A comprehensive review of genetic and epigenetic mechanisms that regulate BDNF expression and function with relevance to major depressive disorder. Am. J. Med. Genet. B Neuropsychiatr. Genet..

[40] Autry A.E., Monteggia L.M. (2012). Brain-derived neurotrophic factor and neuropsychiatric disorders. Pharmacol. Rev..

[41] Fumery M., Singh S., Dulai P.S., Gower-Rousseau C., Peyrin-Biroulet L., Sandborn W.J. (2018). Natural history of adult ulcerative colitis in population-based cohorts: A systematic review. Clin. Gastroenterol. Hepatol..

[42] Fairbrass K.M., Gracie D.J., Ford A.C. (2022). Relative contribution of disease activity and psychological health to prognosis of inflammatory bowel disease during 6.5 years of longitudinal follow-up. Gastroenterology.

[43] Nakase H., Sato N., Mizuno N., Ikawa Y. (2022). The influence of cytokines on the complex pathology of ulcerative colitis. Autoimmun. Rev..

[44] Moulton C.D., Malys M., Hopkins C.W.P., Rokakis A.S., Young A.H., Powell N. (2024). Activation of the interleukin-23/Th17 axis in major depression: A systematic review and meta-analysis. Eur. Arch. Psychiatry Clin. Neurosci..

[45] Schmitz C.N., Sammer G., Neumann E., Blecker C., Gründer G., Adolphi H., Lamadé E.K., Pedraz-Petrozzi B. (2025). Functional resting state connectivity is differentially associated with IL-6 and TNF-alpha in depression and in healthy controls. Sci. Rep..

[46] Reyes-Martínez S., Segura-Real L., Gómez-García A.P., Tesoro-Cruz E., Constantino-Jonapa L.A., Amedei A., Aguirre-García M.M. (2023). Neuroinflammation, microbiota-gut-brain axis, and depression: The vicious circle. J. Integr. Neurosci..

[47] Menard C., Pfau M.L., Hodes G.E., Kana V., Wang V.X., Bouchard S., Takahashi A., Flanigan M.E., Aleyasin H., LeClair K.B. (2017). Social stress induces neurovascular pathology promoting depression. Nature Neurosci..

[48] Ye M., Ji F., Huang C., Li F., Zhang C., Zhang Y., Wang R., Ma K., Lu X., Wang H. (2024). A novel probiotic formula, BLLL, ameliorates chronic stress-induced depression-like behaviors in mice by reducing neuroinflammation and in-creasing neurotrophic factors. Front. Pharmacol..

[49] Benson S., Engler H., Wegner A., Rebernik L., Spreitzer I., Schedlowski M., Elsenbruch S. (2017). What Makes You Feel Sick After Inflammation? Predictors of Acute and Persisting Physical Sickness Symptoms Induced by Experimental Endotoxemia. Clin. Pharmacol. Ther..

[50] Maes M., Kubera M., Leunis J.C. (2008). The gut-brain barrier in major depression: Intestinal mucosal dysfunction with an increased translocation of LPS from gram negative enterobacteria (leaky gut) plays a role in the inflammatory pathophysiology of depression. Neuro Endocrinol. Lett..

[51] Harnik S., Ungar B., Loebstein R., Ben-Horin S. (2024). A gastroenterologist’s guide to drug interactions of small molecules for inflammatory bowel disease. United Eur. Gastroenterol. J..

[52] Ehrhardt M., Schreiber S., Duderstadt Y., Braun-Dullaeus R., Borucki K., Brigadski T., Müller N.G., Leßmann V., Müller P. (2024). Circadian rhythm of brain-derived neurotrophic factor in serum and plasma. Exp. Physiol..

[53] Kanabrocki E.L., Sothern R.B., Ryan M.D., Kahn S., Augustine G., Johnson C., Foley S., Gathing A., Eastman G., Friedman N. (2008). Circadian characteristics of serum calcium, magnesium and eight trace elements and of their metallo-moieties in urine of healthy middle-aged men. Clin. Ter..

[54] Masood T., Kushwaha R.S., Singh R., Sailwal S., Pandey H., Varma A., Singh R.K., Cornelissen G. (2015). Circadian rhythm of serum 25 (OH) vitamin D, calcium and phosphorus levels in the treatment and management of type-2 diabetic patients. Drug. Discov. Ther..

